# Unsupervised Phenotyping of Asthma: Integrating Serum Periostin with Clinical and Inflammatory Profiles

**DOI:** 10.3390/diagnostics15233028

**Published:** 2025-11-27

**Authors:** Sukanya Ravindran, Mohammed Kaleem Ullah, Medha Karnik, Mandya Venkateshmurthy Greeshma, Nidhi Bansal, Shreedhar Kulkarni, Rekha Vaddarahalli ShankaraSetty, SubbaRao V. Madhunapantula, Jayaraj Biligere Siddaiah, Sindaghatta Krishnarao Chaya, Komarla Sundararaja Lokesh, Swaroop Ramaiah, Sachith Srinivas, Vikhnesh Padmakaran, Malavika Shankar, Ashwaghosha Parthasarathi, Padukudru Anand Mahesh

**Affiliations:** 1 Department of Respiratory Medicine, JSS Medical College, JSS Academy of Higher Education & Research, Mysuru 570015, India; sukanya.hema29may@gmail.com (S.R.); greeshmagreekz27@gmail.com (M.V.G.); dr.nidzz2117@gmail.com (N.B.); shreedhar.k.kulkarni@gmail.com (S.K.); rekha.setty23@gmail.com (R.V.S.); bsjayaraj@jssuni.edu.in (J.B.S.); chaya.sindaghatta@gmail.com (S.K.C.); lokeshpulmo@gmail.com (K.S.L.); 2 Center of Excellence in Molecular Biology and Regenerative Medicine (CEMR) Laboratory (DST-FIST Supported Center and ICMR Collaborating Center of Excellence—ICMR-CCoE), Department of Biochemistry (DST-FIST Supported Department), JSS Medical College, JSS Academy of Higher Education & Research, Mysuru 570015, India; ka7eem@gmail.com (M.K.U.); medhakarnik07@gmail.com (M.K.); mvsstsubbarao@jssuni.edu.in (S.V.M.); 3 Thomas Memorial Hospital, WVU Medicine, South Charleston, WV 25303, USA; swaroop2408@gmail.com; 4 Respiratory Medicine, Barnsley NHS Foundation Trust, Barnsley S75 2EP, UK; sachithgowda043@gmail.com; 5 General Medicine, Barnsley NHS Foundation Trust, Barnsley S75 2EP, UK; drvikhneshpadmakaran@gmail.com; 6 Bridgeport Hospital, Yale New Haven Health, Bridgeport, CT 06610, USA; malavika18.shankar@gmail.com; 7 Rutgers RWJ Barnabas Center for Climate, Health and Healthcare, New Brunswick, NJ 08901, USA; ashwa.partha@gmail.com

**Keywords:** asthma, serum periostin, biomarker, asthma phenotypes, unsupervised clustering, disease severity

## Abstract

**Background/Objectives:** Asthma is a heterogeneous inflammatory airway disease. Periostin, a matricellular protein induced by interleukin-13, contributes to airway inflammation and remodeling. This study evaluated serum periostin as a diagnostic biomarker and explored multidimensional phenotypes in adult asthma. **Methods:** A cross-sectional study included 76 adults, with 25 healthy controls, 25 moderate, and 26 severe asthma patients, classified per Global Initiative for Asthma (GINA)-2020 guidelines. Serum periostin was measured using an enzyme-linked immunosorbent assay (ELISA). Diagnostic accuracy was assessed using receiver operating characteristic (ROC) analysis, Firth-penalized logistic regression, bootstrap calibration (1000 resamples), decision curve analysis (DCA), and gradient boosting machine (GBM) validation. Principal component analysis (PCA) followed by k-means clustering identified distinct phenotypes based on clinical, functional, and inflammatory variables. **Results:** Asthma patients had higher serum periostin than controls (median 52.9 vs. 32.5 pg/mL; *p* < 0.01), with excellent diagnostic accuracy (AUC = 0.987; sensitivity = 94.1%, specificity = 100%). Firth regression identified periostin as the only independent predictor of asthma diagnosis (β = 0.387; OR = 1.47; 95% CI 1.23–2.08; *p* < 0.001). Calibration showed minimal error (MAE = 0.042) and DCA demonstrated clear net benefit. GBM confirmed periostin as the dominant diagnostic predictor. PCA revealed three clusters: Cluster 1: younger, lower periostin, preserved lung function, good symptom control; Cluster 2: intermediate periostin, greater airflow limitation, poorer control; and Cluster 3: highest periostin, elevated systemic inflammation (NLR, PLR, SII), with moderate functional impairment. **Conclusions:** Serum periostin is a reliable diagnostic biomarker for asthma. Multidimensional clustering highlights clinically relevant phenotypes linked to periostin, inflammatory burden, and lung function, supporting its role in personalized asthma management.

## 1. Introduction

Asthma is a chronic, heterogeneous respiratory disease characterized by airway inflammation; episodic symptoms including wheezing, breathlessness, chest tightness, and cough; and variable airflow obstruction [1]. Symptoms and airway changes fluctuate in severity and frequency, often triggered by physical exertion, allergen exposure, irritants, environmental changes, and viral infections [1,2,3,4]. Despite advances, asthma remains a significant health burden, particularly in low- and middle-income countries (LMICs) such as India.

In India, asthma affects approximately 34.3 million individuals, representing 13.1% of the global asthma burden [5]. The disease is associated with substantial morbidity and mortality, with a death rate of 13.2 per 100,000 and accounting for 27.9% of disability-adjusted life years (DALYs) in the country [5]. Alarmingly, India’s mortality rate is three times the global average, with nearly twice as many DALYs attributable to asthma [5]. Globally, asthma prevalence and mortality have decreased by 24% and 51%, respectively, since 1990; however, the disease still accounts for considerable health loss, with a 2019 DALY rate of 273.6 [6].

Airway remodeling a key pathological feature of asthma involves basement membrane thickening and excessive deposition of extracellular matrix (ECM) proteins such as collagen, elastin, and fibronectin [7]. Among these, periostin, a matricellular ECM protein, plays a central role in tissue repair and remodeling. Normally expressed at low levels, periostin is upregulated during inflammation and fibrosis [7].

Periostin expression is induced in airway epithelial cells by Th2 cytokines, particularly interleukin (IL)-4 and IL-13, pivotal mediators of type 2 inflammation in asthma [7]. It facilitates eosinophil recruitment, migration, and adhesion within the airway matrix, exacerbating eosinophilic inflammation [7]. Moreover, periostin promotes airway remodeling by stimulating fibroblast-mediated collagen deposition via transforming growth factor beta (TGF-β) signaling, increasing airway stiffness, and contributing to irreversible airflow limitation [8,9].

Given periostin’s integral role in asthma pathophysiology, it has emerged as a promising biomarker reflecting airway inflammation and remodeling. However, data on serum periostin levels remain limited for adult populations, especially in LMICs, where environmental and demographic factors may alter disease expression [10,11,12]. Diagnosing asthma is often challenging, as patients may have normal lung function during assessments, and standard spirometric criteria frequently fail to confirm diagnosis. Studies have shown low concordance between clinical asthma diagnosis and objective reversibility testing, with only 10–16% confirmation rates in some cohorts [13,14,15].

Beyond diagnosis, periostin holds potential in guiding asthma phenotyping, helping identify patients with Th2-high inflammation who may benefit from targeted biologics (e.g., anti-IL-13 and anti-IL-4 receptor therapies) [16,17,18,19]. Elevated periostin also correlates with asthma exacerbation risk and may serve as a marker of ongoing airway remodeling [20]. Incorporating periostin measurement into clinical algorithms can enhance precision medicine approaches, tailoring therapy to individual patient profiles. Considering these factors, investigating serum periostin’s diagnostic and prognostic capabilities in adult Indian patients is crucial. This study aims to evaluate serum periostin’s utility in diagnosing asthma and distinguishing disease severity in a South Indian cohort, contributing valuable insights for resource-limited settings.

## 2. Materials and Methods

### 2.1. Study Population

We conducted a cross-sectional Case–Control study at the Department of Respiratory Medicine in a university-affiliated 1800-bed tertiary care hospital in South India from 1 January 2021 to 30 June 2022. The study protocol was approved by the Institutional Ethics Committee of the Medical College, Mysuru (Approval number: JSS/MC/PG/5156/2020-21). Written informed consent was obtained from all participants or their legal guardians.

Adult patients (age ≥ 18 years) diagnosed with asthma according to the Global Initiative for Asthma (GINA) 2020 guidelines [1] attending the outpatient department or admitted to the hospital were recruited. A total of 76 participants were included: 51 asthma patients (26 severe asthma and 25 moderate asthma) and 25 healthy controls. Exclusion criteria included lack of consent, COPD or other chronic respiratory/atopic diseases known to elevate serum periostin (such as atopic dermatitis, interstitial lung disease, metastatic bone disease), age below 18 or above 65 years, major systemic illnesses, pregnancy, and patients previously misdiagnosed or treated as asthma without confirmatory evidence (asthma mimickers). Diagnosis was confirmed per GINA 2020 guidelines [1] using spirometry according to American Thoracic Society (ATS) standards [21].

Detailed demographic and clinical data were collected, including age, sex, body mass index (BMI), smoking history, and medical history. Asthma severity classification was based on the National Asthma Education and Prevention Program (NAEPP) guidelines [22]. Healthy controls, recruited from the community, had no respiratory symptoms, normal spirometry, and no history of eczema, allergic rhinitis, or other atopic disorders.

### 2.2. Detection of Serum Periostin

Venous blood (5 mL) was collected by venipuncture from each participant. Samples were allowed to clot at room temperature for 30 min and then centrifuged, and serum was aliquoted and stored at −80 °C until assay. Serum periostin levels were measured using a commercially available enzyme-linked immunosorbent assay (ELISA) kit (Cat No. CK-bio-12826, Shanghai Coon Koon Biotech Co., Ltd., Shanghai, China). The assay employed a quantitative sandwich ELISA format with precoated human periostin-specific monoclonal antibodies on 96-well plates. After sample incubation and washing steps, the enzymatic substrate reaction was performed, and the optical density was read at the recommended wavelength using an automated plate reader.

Complete blood counts were performed, and inflammatory indices, including the neutrophil-to-lymphocyte ratio (NLR) and platelet-to-lymphocyte ratio (PLR), were calculated as part of systemic inflammation assessment.

In addition, the Systemic Immune-Inflammation Index (SII) was calculated to provide a composite measure of systemic inflammatory status. SII was computed using the formula SII = (Neutrophils × Platelets)/Lymphocytes.

### 2.3. Statistical Analysis

Data analysis was conducted using jamovi version 2.7.6 (The jamovi Project, Sydney, Australia) and R version 4.5.1 within RStudio version 2025.09.1+401 (Posit Software, Boston, MA, USA). Descriptive statistics characterized the study population. Continuous variables were examined for normality using the Shapiro–Wilk test. Normally distributed data were expressed as mean ± standard deviation, and non-normal data as median (interquartile range). Between-group comparisons utilized Student’s *t*-test or Mann–Whitney U test for continuous variables, and chi-square test for categorical variables. Correlations were assessed by Pearson’s r for normally distributed and Spearman’s rho for non-normal data.

Receiver operating characteristic (ROC) curve analysis was conducted to determine the diagnostic performance of serum periostin, calculating the area under the curve (AUC), sensitivity, specificity, and optimal cut-off values using Youden’s index. Statistical significance threshold was set at *p* < 0.05 (two-tailed).

To address potential small-sample bias and separation, Firth’s penalized logistic regression was performed (logistf package v1.26.1), with odds ratios, profile-likelihood confidence intervals, and penalized *p*-values reported. Model performance and validity were evaluated using complementary approaches. Calibration was examined through 1000-bootstrap resampling (rms package v8.1.0) to compare predicted and observed probabilities. Clinical utility was assessed using decision curve analysis (DCA) (rmda package v1.6), comparing the model’s net benefit across threshold probabilities with “treat-all” and “treat-none” strategies. To mitigate overfitting concerns, internal stability was tested using a gradient boosting machine (GBM v2.2.2) with repeated 10-fold cross-validation (caret package v7.0.1), and variable-importance scores were used to confirm periostin as the dominant predictor.

Covariates were included based on their known clinical relevance to asthma and periostin levels. Age [23,24], gender [24,25], and BMI [26,27] influence inflammatory responses and periostin expression. Asthma severity and systemic inflammatory markers (NLR [28], PLR [29], eosinophils [30], SII [31]) were included to adjust for disease-related inflammatory variability and reduce confounding.

To explore underlying patterns and identify potential patient subgroups within the cohort, multivariate analyses were conducted. Principal component analysis (PCA) was planned to reduce dimensionality across clinical, spirometric, serum periostin, and inflammatory biomarker variables. Retained components for further analysis were to be determined based on eigenvalue and scree plot criteria. Subsequently, unsupervised k-means clustering was applied using standardized principal component scores to detect latent phenotypic clusters in the dataset. The optimal number of clusters was evaluated using the elbow method and inspection of the within-cluster sum of squares. Centroid profiles and variable contribution plots were generated for the characterization and visualization of each cluster. These methods were intended to supplement traditional univariate comparisons and facilitate recognition of clinically meaningful, multidimensional asthma phenotypes within the study population.

## 3. Results

**Figure 1:**
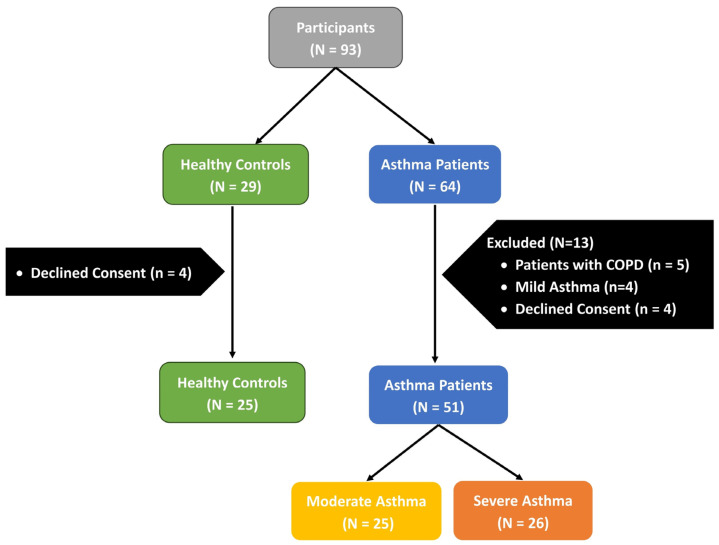
Study flowchart depicting subject recruitment.

A total of 76 adult participants were enrolled, including 51 asthma patients (26 severe, 25 moderate) and 25 healthy controls (Figure 1). Severe asthma patients were significantly older than moderate asthma patients and controls [45.5 (34.8–56.1) vs. 30.5 (23.0–46.0) and 36.0 (26.3–45.0) years; *p* < 0.001]. Gender distribution and BMI did not differ significantly among the three groups (*p* = 0.880 and *p* = 0.560, respectively). Serum periostin levels were significantly elevated in both asthma groups compared with healthy controls [moderate: 51.1 (48.8–52.8) pg/mL; severe: 52.5 (48.6–56.4) pg/mL vs. controls: 35.5 (25.1–40.8) pg/mL; *p* < 0.001]. Pulmonary function tests showed that severe asthma patients demonstrated markedly reduced pre-bronchodilator FEV_1_ % predicted (56.0%) compared to moderate asthma (80.0%) and healthy controls (80.0%) (*p* < 0.001). Predicted pre-bronchodilator FVC% was also significantly lower in severe asthma (69.5%) than in moderate asthma (90.0%) and controls (81.0%) (*p* < 0.001). Additionally, the pre-bronchodilator FEV_1_/FVC ratio was reduced in severe asthma (79.0%) compared with moderate asthma (88.0%) and controls (81.6%) (*p* < 0.001). Post-bronchodilator values continued to show impairment in severe asthma, with lower FEV_1_ (68.5% vs. 91.0% in moderate asthma; *p* < 0.001) and FEV_1_/FVC ratio (87.5% vs. 96.0%; *p* < 0.001). No significant differences were observed in smoking status, disease duration, inflammatory markers (NLR, PLR), or eosinophil percentages between moderate and severe groups. Asthma control test (ACT) scores were lower in severe asthma, reflecting poorer disease control (13.0 vs. 17.0, *p* < 0.01) (Table 1).

**Figure 2:**
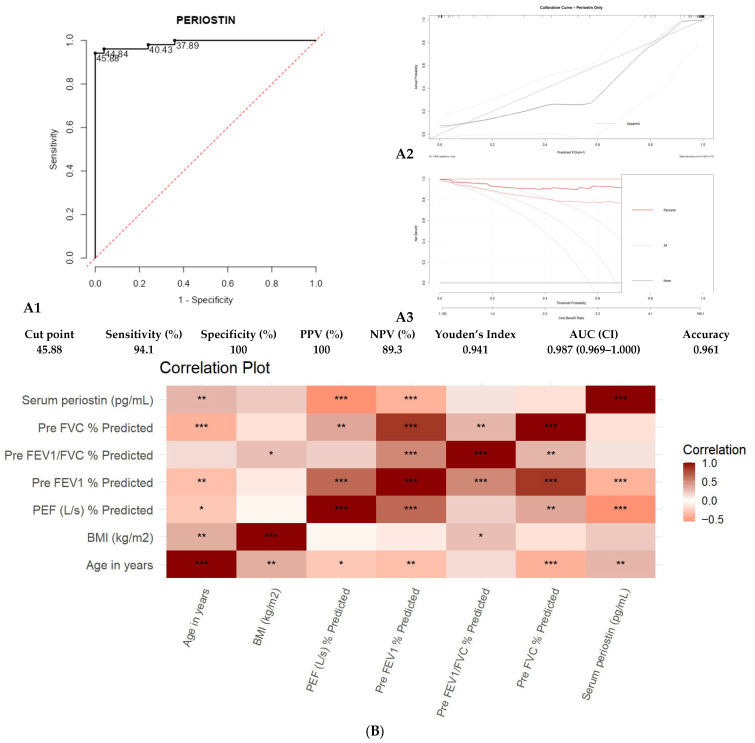
(**A1**) shows the ROC curve of serum periostin levels for predicting asthma (controls vs. cases), (**A2**) presents the calibration curve for the periostin-only model and (**A3**) displays the DCA showing the net clinical benefit across threshold probabilities. (**B**) Heat Map depicting the Correlation Matrix of all variables of controls and asthma subjects. Note: FVC: Forced Vital Capacity; FEV_1_: Forced Expiratory Volume in 1 s; PEF: Peak Expiratory Flow; pg/mL: picograms per milliliter; BMI: Body mass index; kg/m^2^: kilogram per square meter. * = *p* < 0.05, ** = *p* < 0.01, *** = *p* < 0.001.

Receiver operating characteristic (ROC) curve analysis revealed that serum periostin > 45.88 pg/mL robustly discriminated asthma patients from controls with 94.1% sensitivity, 100% specificity, 100% positive predictive value, 89.3% negative predictive value, and an AUC of 0.987 (Figure 2A1).

The periostin-only logistic model showed good calibration on 1000-bootstrap resampling, with a mean absolute calibration error of 0.042 and an optimism-corrected calibration slope of 0.88, indicating minimal overfitting (Figure 2A2; Supplementary Table S1). DCA demonstrated higher net clinical benefit than treat-all or treat-none strategies across threshold probabilities of 5–70% (Figure 2A3). Internal stability assessment using gradient boosting confirmed periostin as the dominant predictor, with substantially lower importance for all other covariates (Supplementary Table S2).

A significant positive correlation was found between serum periostin levels and age (r = 0.300, *p* < 0.01) and borderline with BMI (r = 0.225, *p* = 0.051), while periostin inversely correlated with pre-FEV1% predicted (r = −0.394, *p* < 0.001) (Figure 2B). Pre-FEV1% and pre-FVC % predicted declined with age, consistent with disease progression.

Serum periostin levels were significantly higher in asthma patients compared with healthy controls. In the Firth-penalized logistic regression model, periostin was the only significant predictor of asthma diagnosis (β = 0.387, OR = 1.47; 95% CI: 1.23–2.08; *p* < 0.001). Age, gender, BMI, smoking status, and pre-bronchodilator FEV_1_% predicted were not significant contributors (*p* > 0.05) (Table 2).

**Figure 3:**
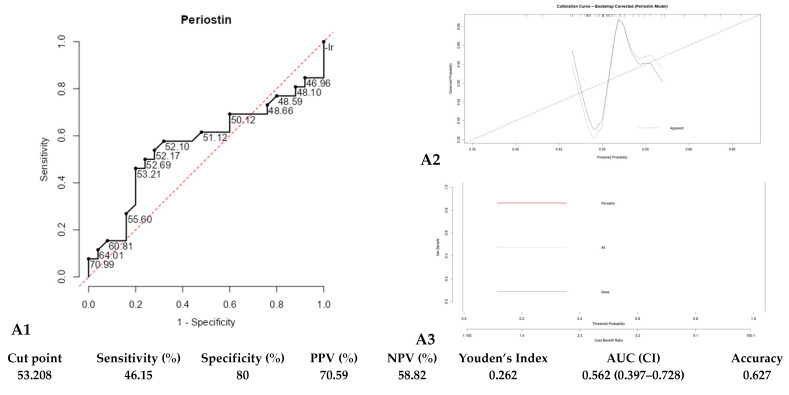

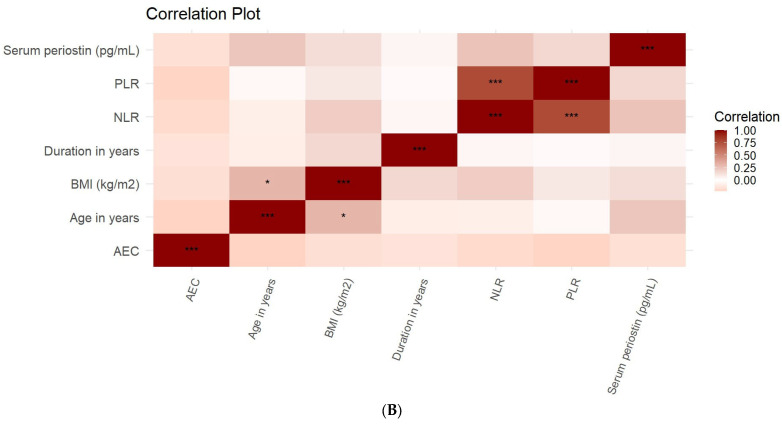
(**A1**) shows the ROC curve of serum periostin levels for predicting asthma severity (moderate vs. severe), (**A2**) presents the calibration curve for the periostin-only model and (**A3**) displays the DCA showing the net clinical benefit across threshold probabilities. (**B**) Heat Map depicting the Correlation Matrix of all variables of Moderate and Severe asthma cases. Notes: NLR: Neutrophil-to-Lymphocyte ratio; PLR: Platelet-to-Lymphocyte Ratio; pg/mL: picograms per milliliter; BMI: Body mass index; kg/m^2^: kilogram per square meter; AEC: Absolute Eosinophil Count. * = *p* < 0.05, *** = *p* < 0.001.

In distinguishing asthma severity, ROC analysis showed limited discriminatory power for periostin (cut-off of 53.208 pg/mL, sensitivity of 46.2%, specificity of 80.0%, AUC of 0.562) (Figure 3A1). The periostin-only model showed poor discrimination for predicting asthma severity (AUC = 0.56). Bootstrap calibration (1000 resamples) demonstrated weak model fit, with a low optimism-corrected calibration slope (0.57) and a mean absolute calibration error of 0.069, indicating limited predictive reliability (Figure 3A2; Supplementary Table S3). DCA showed no meaningful net clinical benefit, with the periostin curve overlapping the treat-all and treat-none strategies (Figure 3A3). Gradient boosting confirmed that periostin had minimal predictive importance, far lower than age, SII, BMI, and PLR (Supplementary Table S4).

Within moderate and severe groups, serum periostin showed positive but non-significant correlations with BMI, disease duration, NLR, and PLR and inverse non-significant correlations with ACT scores and absolute eosinophil counts (Figure 3B).

Serum periostin levels did not differ significantly between patients with severe and moderate asthma. In the Firth-penalized logistic regression model, age was the only significant predictor of asthma severity (β = 0.067, OR = 1.07; 95% CI: 1.02–1.14; *p* = 0.008). Periostin, gender, BMI, NLR, PLR, SII, and eosinophilia were not significant predictors in the multivariable model (all *p* > 0.05) (Table 3).

**Figure 4:**
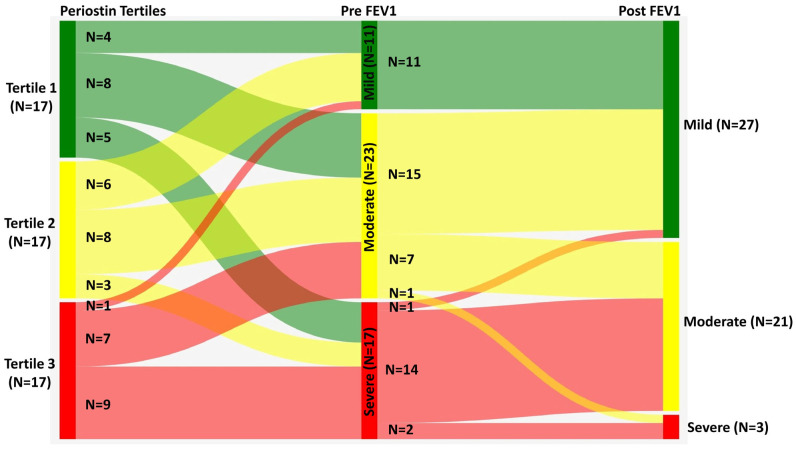
Sankey chart depicting the association between periostin tertiles and the severity of airflow limitation (pre- and post-bronchodilator) among adolescents with asthma in study participants.

Sankey diagram provides insight into the relationship between serum periostin levels and spirometric reversibility: individuals with lower baseline FEV_1_ (<60% predicted) and high or intermediate periostin levels generally shifted to the 60–80% post-bronchodilator FEV_1_ range, indicating substantial reversibility. In contrast, those with normal or near-normal baseline FEV_1_ (>80%) and low periostin levels remained largely unchanged after bronchodilation. These visualized transitions suggest that high periostin is most strongly associated with impaired baseline lung function and marked, but not complete, reversibility, rather than with fixed or non-reversible airflow limitation (Figure 4).

**Figure 5:**
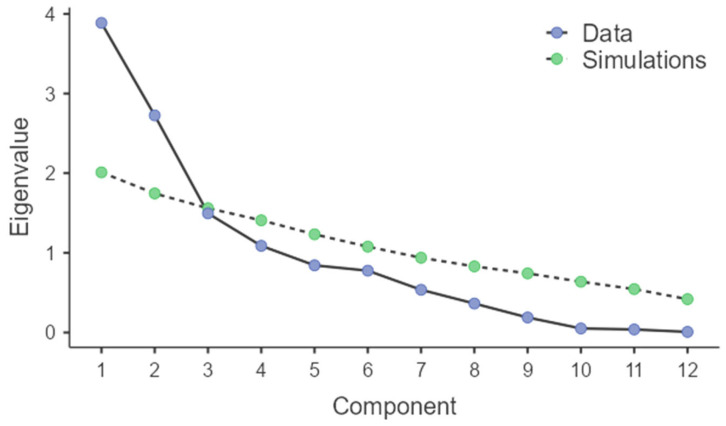
Scree plot showing the eigenvalues for principal components. The plot depicts the eigenvalues of the observed data (blue) and simulated data (green) for each principal component. The first three components account for the majority of the variance in the dataset, justifying their use for subsequent k-means clustering to identify distinct asthma phenotypes.

**Figure 6:**
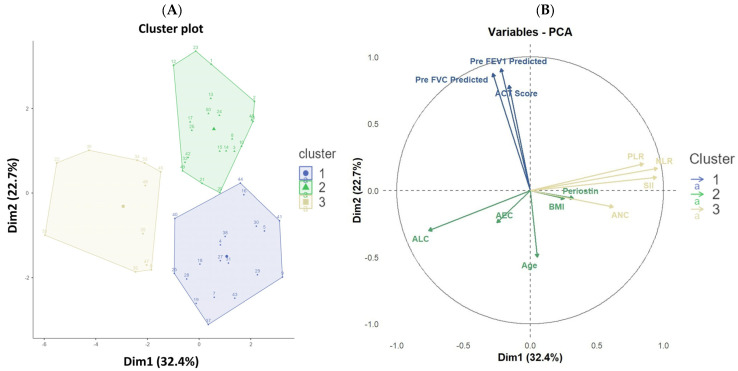
K-means cluster plot of asthma participants based on multidimensional variables (**A**) and PCA biplot of quantitative variables (**B**). Note: FVC: Forced Vital Capacity; FEV_1_: Forced Expiratory Volume in 1 Second; BMI: Body mass index; ACT: Asthma Control Test; NLR: Neutrophil-to-Lymphocyte ratio; PLR: Platelet-to-Lymphocyte Ratio; AEC: Absolute Eosinophil Count; ANC: Absolute Neutrophil Count; ALC: Absolute Lymphocyte Count; SII: Systemic Immune-Inflammation Index; PCA: Principal component analysis.

Principal component analysis (PCA) was performed to reduce dimensionality and explore underlying patterns among the variables in our study cohort. The first three principal components accounted for 67.6% of the total variance in the data, as indicated by the eigenvalues (Table 4) and scree plot (Figure 5). Utilizing these components, unsupervised k-means clustering was applied, revealing three distinct patient clusters within the cohort. Cluster 1 (*n* = 19) was characterized by below-average age, BMI, and periostin, but showed notably higher ACT scores and better spirometric indices (pre-FEV_1_ and pre-FVC predicted), suggesting a cluster of patients with relatively preserved lung function and good asthma control. Cluster 2 (*n* = 21) included patients with above-average age with mostly severe asthma, lower ACT scores, and spirometric values compared to Cluster 1, indicating older patients with poorer asthma control and reduced lung function. Cluster 3 (*n* = 11) was marked with intermediate age (median 40 years), mixed asthma severity, highest periostin levels (median 53.1 pg/mL), elevated BMI, and pronounced systemic inflammation (NLR 5.1, PLR 200, SII 1247). ACT scores and spirometric values were intermediate (pre-FEV1 65%, pre-FVC 79%), representing a phenotype with increased type 2-driven and systemic inflammatory burden. Eosinophilia was present in 72.7% of patients (Table 5). This cluster may represent a distinct phenotype with pronounced type 2 inflammation and systemic inflammatory burden. The optimal number of clusters was validated both by inspection of the scree plot and the k-means elbow method. Biplot visualization and cluster plots further confirmed distinct groupings among subjects (Figure 6). Together, these analyses highlight that multidimensional unsupervised clustering can identify clinically relevant asthma phenotypes based on combined periostin, inflammatory, demographic, and functional parameters within this population.

**Table 4:** Eigenvalues, Percentage of Variance, and Cumulative Variance for Principal Components.

Component	Eigenvalue	% of Variance	Cumulative %
1	3.887	32.395	32.395
2	2.725	22.712	55.107
3	1.495	12.454	67.561
4	1.087	9.061	76.623
5	0.843	7.024	83.647
6	0.776	6.466	90.113
7	0.536	4.467	94.580
8	0.364	3.031	97.611
9	0.189	1.574	99.185
10	0.051	0.427	99.611
11	0.038	0.320	99.932
12	0.008	0.068	100.000

**Table 5:** Demographic data characteristics among different clusters.

	Cluster 1 (*N* = 19)	Cluster 2 (*N* = 21)	Cluster 3 (*N* = 11)	*p*-Value
Age in years	27.0 (23.0–44.0)	47.0 (35.7–57.0)	40.0 (25.3–47.0)	0.001 ^1^
Gender				0.527 ^2^
Female (*n*, %)	7.0 (36.8%)	11.0 (52.4%)	6.0 (54.5%)	
Male (*n*, %)	12.0 (63.2%)	10.0 (47.6%)	5.0 (45.5%)	
Severity				<0.001 ^2^
Moderate	19.0 (100.0%)	1.0 (4.8%)	5.0 (45.5%)	
Severe	0.0 (0.0%)	20.0 (95.2%)	6.0 (54.5%)	
Periostin(pg/mL)	50.9 (49.0–52.5)	51.2 (48.0–53.9)	53.1 (52.1–58.6)	0.210 ^1^
BMI (kg/m^2^)	25.1 (19.5–30.7)	24.1 (20.4–26.8)	24.2 (22.0–28.4)	0.720 ^1^
Smoking				0.374 ^2^
No	16.0 (84.2%)	19.0 (90.5%)	11.0 (100.0%)	
Yes	3.0 (15.8%)	2.0 (9.5%)	0.0 (0.0%)	
Duration in years	3.0 (1.0–6.0)	2.0 (0.8–3.0)	3.0 (1.0–5.7)	0.210 ^1^
ACT Score	17.0 (16.0–18.8)	13.0 (12.0–14.0)	13.0 (14.0–16.8)	<0.001 ^1^
NLR	2.3 (1.4–3.1)	2.0 (1.6–2.9)	5.1 (4.8–6.5)	<0.001 ^1^
PLR	107.9 (75.4–129.9)	100.0 (82.9–141.1)	200.0 (173.2–225.8)	<0.001 ^1^
SII	518.3 (393.7–631.3)	516.4 (428.1–697.8)	1247.5 (1131.2–1784.5)	<0.001 ^1^
Eosinophilia				0.260 ^2^
No	7.0 (36.8%)	3.0 (14.3%)	3.0 (27.3%)	
Yes	12.0 (63.2%)	18.0 (85.7%)	8.0 (72.7%)	
Pre-FVC predicted	92.0 (87.0–98.8)	72.0 (63.7–75.3)	79.0 (68.2–83.8)	<0.001 ^1^
Pre-FEV_1_ predicted	81.0 (72.7–85.8)	56.0 (45.0–60.3)	65.0 (56.2–73.7)	<0.001 ^1^
Post-FVC predicted	98.0 (91.2–105.8)	80.0 (73.0–89.7)	87.0 (84.2–90.8)	<0.001 ^1^
Post-FEV_1_ predicted	94.0 (85.3–102.2)	68.0 (63.3–77.3)	83.0 (70.3–87.2)	<0.001 ^1^
Pre-FEV_1_/FVC predicted	88.0 (82.0–90.0)	79.0 (71.7–84.0)	85.0 (78.0–89.7)	<0.001 ^1^
Post-FEV_1_/FVC predicted	95.0 (91.0–98.7)	88.0 (83.0–91.0)	92.0 (86.3–96.8)	<0.001 ^1^
AEC	490.0 (241.7–735.0)	560.0 (400.0–820.0)	500.0 (216.7–641.7)	0.400 ^1^
ANC	4790.0 (3755.0–5818.3)	5160.0 (4366.7–5656.7)	6780 (5888.3–7816.7)	<0.001 ^1^
ALC	2260.0 (1468.3–3156.7)	2400.0 (1813.3–3100.0)	1280.0 (1196.7–1320.0)	<0.001 ^1^

^1^ Kruskal-Wallis. ^2^ Pearson. pg/mL: picograms per milliliter; BMI: Body mass index; kg/m^2^: kilogram per square meter; ACT: Asthma Control Test; NLR: Neutrophil-to-Lymphocyte ratio; PLR: Platelet-to-Lymphocyte Ratio; SII: Systemic Immune-Inflammation Index; FVC: Forced Vital Capacity; FEV_1_: Forced Expiratory Volume in 1 s; AEC: Absolute Eosinophil Count; ANC: Absolute Neutrophil Count; ALC: Absolute Lymphocyte Count.

## 4. Discussion

This cross-sectional study confirms that serum periostin is a robust and reliable biomarker for asthma diagnosis among adults in a South Indian cohort. We demonstrated significantly elevated serum periostin levels in asthmatics compared to healthy controls, with a diagnostic threshold of 45.95 pg/mL yielding excellent accuracy (94.1% sensitivity; 100% specificity; AUC 0.987). Importantly, serum periostin remained an independent predictor of asthma after adjusting for relevant confounders, including age, sex, and lung function metrics. Each 1 pg/mL rise in periostin increases the log-odds of asthma by 0.387; therefore, a 10 pg/mL elevation, well within the typical biological range, would increase the odds of asthma by almost four-fold. Conversely, periostin failed to distinguish between moderate and severe asthma, indicating its limitation for severity classification within this population. In exploratory analyses, unsupervised multivariate approaches elaborated on the heterogeneity within the cohort. Principal component analysis combined clinical, spirometric, and inflammatory biomarkers, including periostin, identifying key axes of variation. Subsequent k-means clustering revealed three distinct patient clusters, characterized by differing profiles of age, BMI, periostin levels, lung function (FEV_1_ and FVC % predicted), and inflammatory cell counts (ANC, ALC, NLR, PLR, SII). Notably, the third cluster demonstrated slightly elevated periostin alongside and significantly elevated systemic inflammation markers, coupled with intermediate lung function, suggesting a distinct phenotype not captured by conventional severity classifications.

The observed diagnostic performance aligns well with the broader literature. Seminal work by Izuhara et al. first established periostin’s elevation in asthma, with mean serum levels approximately threefold higher in patients versus controls (86.0 vs. 33.0 ng/mL) [9]. Subsequent meta-analyses have confirmed these findings, indicating that elevated serum periostin confers an increased odds ratio for asthma diagnosis ranging from approximately 8 to 14 [32,33]. Our results add important validation from a South Asian, LMIC context, an essential contribution given the prior overrepresentation of Western populations in biomarker studies.

Periostin’s biological role contextualizes these diagnostic associations. It is an extracellular matrix protein upregulated in response to IL-4 and IL-13, signaling-key drivers of type 2 (T2) inflammation characterizing a major asthma endotype [7,8]. This T2-high phenotype is typified by eosinophilic airway infiltration and pronounced tissue remodeling, where periostin facilitates subepithelial fibrosis by promoting collagen deposition mediated via TGF-β pathways [8,34]. Our data, demonstrating correlations of periostin with advancing age and declining pulmonary function, particularly reduced FEV_1_ and FEV_1_/FVC ratios, further support periostin as a marker of progressive airway remodeling and functional decline [35,36].

While periostin’s utility as a diagnostic biomarker for asthma is well supported, its performance for disease severity stratification remains inconsistent across studies. Similarly to our findings, large cohorts have found no significant periostin differences across GINA severity classifications [27,37]. In contrast, select investigations describe increasing periostin levels upon worsening asthma severity or poor disease control [38,39,40], albeit often confounded by heterogeneous patient selection or co-existing comorbidities. Our inability to identify periostin as a severity biomarker may reflect such heterogeneity, limited sample size, or methodological differences. Additionally, factors including smoking and obesity, prevalent in our cohort and known to modulate periostin levels inversely [27,41,42,43], potentially attenuate these associations.

Beyond diagnosis and severity, periostin’s most transformative impact lies in its integration into asthma phenotyping and personalized management. Clinical trials of biologic therapies, notably anti-IL-4Rα agents like dupilumab and anti-IL-13 therapies such as lebrikizumab, have shown marked efficacy among patients with elevated periostin reflecting active T2 inflammation and airway remodeling [44,45]. Thus, periostin measurement facilitates precision medicine by distinguishing T2-high responders poised to benefit from these costly targeted treatments. Moreover, periostin has emerged as a dynamic biomarker for monitoring disease activity, with elevations presaging exacerbations and supporting preemptive therapeutic intensification [46].

Further utility extends to evaluating remodeling and fixed airflow limitation, as periostin levels correlate inversely with lung function metrics, including post-bronchodilator FEV_1_ and FEV_1_/FVC [38,47]. This suggests potential for periostin to identify patients at risk of irreversible airway obstruction, a critical consideration given that current standard spirometry offers limited insight into ongoing fibrotic progression. Integrating periostin with established biomarkers such as blood eosinophils and Fractional Exhaled Nitric Oxide (FeNO) advances development of comprehensive endotyping algorithms, enabling better patient stratification and tailored interventions [47,48,49]. Our findings varied from the previous literature. The Sankey diagram clearly illustrates dynamic changes in lung function across periostin strata: individuals with higher periostin values (>53.17 pg/mL) disproportionately belonged to the lowest baseline FEV_1_ group (<60% predicted), but the majority in this group showed marked post-bronchodilator improvement, shifting predominantly into the 60–80% predicted range and, to a lesser extent, into the >80% range. By contrast, those with lower periostin levels tended to have better baseline lung function and maintained high post-bronchodilator FEV_1_, suggesting that periostin is associated with more severe but reversible airflow limitation in the cross-sectional setting rather than irreversible fixed obstruction or established airway remodelling. Nevertheless, as this study is cross-sectional and not longitudinal, the ultimate utility of periostin in predicting or tracking airway remodeling and fixed airflow limitation remains to be determined.

Notably, while pediatric data remain sparser, emerging studies confirm periostin’s relevance for asthma diagnosis and monitoring in children, including wheezing phenotypes and response to treatment [39,50,51]. Additionally, periostin’s roles in other type 2-mediated disorders, including chronic rhinosinusitis and atopic dermatitis, open avenues for broader clinical application beyond asthma alone [52,53,54].

PCA in this study revealed that two major dimensions accounted for over half the total variance among asthma cases, effectively separating clinical, functional, and biomarker axes of disease heterogeneity. K-means clustering identified three distinct phenotypic groups: one cluster (Cluster 1) was characterized by lower periostin, younger age, increased BMI, better symptom control (higher ACT scores), and preserved lung function; Cluster 2 showed intermediate periostin but more pronounced airflow limitation and lower ACT scores; and Cluster 3 was notable for the highest periostin, older age, and elevated markers of airway and systemic inflammation (NLR, PLR, SII), with only modest reduction in spirometric indices. Visualization of variable contributions confirms that serum periostin, age, ANC, NLR, PLR, and SII drive much of the variance along the primary axes, while lung function and ACT score delineate the opposite, less inflammatory phenotype. These findings demonstrate a high degree of technical concordance with international cluster analyses that highlight serum periostin as a pivotal biomarker defining severe, type 2–high, and frequently late-onset or obesity-associated asthma endotypes [17,27,55,56]. Similarly, large Asian cohort studies employing unsupervised learning report periostin as a discriminant variable within clusters characterized by persistent airflow limitation, airway structural change, suboptimal symptom control, and heightened exacerbation risk [12,57,58].

Our study’s strengths include rigorous application of well-established diagnostic criteria (GINA, ATS, NAEPP), incorporation of inflammatory indices (NLR, PLR, SII), and recruitment from a representative clinical setting in India. This addresses a gap in biomarker research from low- and middle-income countries where differences in genetics, environmental exposures, and healthcare infrastructure may influence biomarker profiles. The matched healthy control group further solidifies comparative assessments.

Nevertheless, limitations merit consideration. The modest sample size and cross-sectional design impair causal inference and limit the detection of subtler associations, particularly regarding severity. The single-center recruitment from tertiary care may introduce selection bias, potentially limiting generalizability to community-based populations. Absence of longitudinal sampling precluded evaluation of periostin’s temporal dynamics relative to exacerbations or treatment response. Also, local assay methods and ethnic variability must be considered when extrapolating threshold values internationally.

Future research should prioritize prospective longitudinal cohorts integrating periostin with emerging multi-omics signatures and additional biomarkers for nuanced endotyping. Integrating periostin assessments with established biomarkers such as blood eosinophils and FeNO can advance comprehensive endotyping and patient stratification, supporting more personalized interventions in asthma management. Development of cost-effective, point-of-care periostin assays will be pivotal for clinical translation, especially in resource-constrained environments. Together, such advances promise enhanced diagnostic accuracy, refined prognostic stratification, and optimized personalized therapy for global asthma populations.

## 5. Conclusions

Serum periostin demonstrates potential as a diagnostic biomarker for adult asthma in a South Indian cohort, showing high sensitivity and specificity. While it did not robustly differentiate disease severity in our study, further investigation in larger, longitudinal, and multicenter cohorts, including assessments of type 2 biomarkers (FeNO, total IgE) and measures of airway remodeling, is warranted to clarify periostin’s utility in precision asthma management.

## Figures and Tables

**Table 1:** Demographic data characteristics among moderate and severe asthma cases.

	Moderate (*N* = 25)	Severe (*N* = 26)	Healthy Control (*N* = 25)	*p*-Value
Age in years	30.5 (23.0–46.0)	45.5 (34.8–56.1)	36.0 (26.3–45.0)	<0.001 ^1^
Gender				0.880 ^2^
Female (*n*, %)	11.0 (44.0%)	13.0 (50.0%)	14 (56%)	
Male (*n*, %)	14.0 (56.0%)	13.0 (50.0%)	11 (44%)	
Periostin (pg/mL)	51.1 (48.8–52.8)	52.5 (48.6–56.4)	35.5 (25.1–40.8)	<0.001 ^1^
BMI (kg/m^2^)	24.2 (20.0–30.2)	24.8 (20.6–27.3)	24.2 (21.5–24.5)	0.560 ^1^
Smoking				0.605 ^2^
No	22.0 (88.0%)	24.0 (92.3%)	-	
Yes	3.0 (12.0%)	2.0 (7.7%)	-	
Duration in years	3.0 (1.0–4.7)	2.0 (1.0–4.1)	-	0.330 ^1^
ACT Score	17.0 (16.0–18.0)	13.0 (12.0–14.0)	-	<0.001 ^1^
NLR	2.6 (1.8–3.9)	2.5 (1.8–3.6)	-	0.830 ^1^
PLR	112.7 (86.8–171.6)	104.7 (90.0–165.9)	-	0.763 ^1^
SII	541.1 (412.2–829.1)	660.1 (430.4–830.9)	-	0.520 ^1^
Eosinophil %				0.296 ^2^
No	8.0 (32.0%)	5.0 (19.2%)	-	
Yes	17.0 (68.0%)	21.0 (80.8%)	-	
Pre-FVC predicted	90.0 (84.3–97.3)	69.5 (62.8–75.1)	81.0 (65.7–92.3)	<0.001 ^1^
Pre-FEV_1_ predicted	80.0 (71.7–83.0)	56.0 (45.0–60.1)	80.0 (66.3–94.0)	<0.001 ^1^
Post-FVC predicted	97.0 (89.7–105.3)	81.0 (73.8–89.5)	-	<0.001 ^1^
Post-FEV_1_ predicted	91.0 (83.7–97.3)	68.5 (64.9–77.4)	-	<0.001 ^1^
Pre-FEV_1_/FVC predicted	88.0 (82.0–90.0)	79.0 (72.9–84.0)	81.6 (79.3–83.3)	<0.001 ^1^
Post-FEV_1_/FVC predicted	96.0 (91.0–97.7)	87.5 (83.0–91.0)	-	<0.001 ^1^
AEC	420.0 (246.7–650.0)	590.0 (407.5–812.5)	-	0.170 ^1^
ANC	5100.0 (4293.3–6156.7)	5265.0 (4634.2–6500.4)	-	0.570 ^1^
ALC	1830.0 (1333.3–3113.3)	2100.0 (1662.5–2944.2)	-	0.510 ^1^

^1^ Wilcoxon. ^2^ Pearson. pg/mL: picograms per milliliter; BMI: Body mass index; kg/m^2^: kilogram per square meter; ACT: Asthma Control Test; NLR: Neutrophil-to-Lymphocyte ratio; PLR: Platelet-to-Lymphocyte Ratio; SII: Systemic Immune-Inflammation Index; FVC: Forced Vital Capacity; FEV_1_: Forced Expiratory Volume in 1 s; AEC: Absolute Eosinophil Count; ANC: Absolute Neutrophil Count; ALC: Absolute Lymphocyte Count.

**Table 2:** Firth-Penalized Logistic Regression for Predicting Asthma.

				95% CI			
Variable	β (Coefficient)	SE (β)	OR	Lower	Upper	χ^2^	*p*-Value
Periostin (pg/mL)	0.387	0.101	1.473	1.233	2.085	45.724	<0.0001
Age (years)	−0.033	0.038	0.968	0.870	1.056	0.524	0.469
Gender (Female vs. Male)	−0.334	0.963	0.716	0.060	6.538	0.092	0.761
BMI (kg/m^2^)	0.010	0.119	1.010	0.729	1.341	0.005	0.945
Smoker (Yes vs. No)	0.030	1.733	1.030	0.005	42.450	0.0002	0.988
Pre-FEV_1_ % predicted	−0.031	0.028	0.970	0.903	1.033	0.922	0.337

pg/mL: picograms per milliliter; BMI: Body mass index; kg/m^2^: kilogram per square meter; FEV_1_: Forced Expiratory Volume in 1 s; CI: Confidence Interval.

**Table 3:** Firth-Penalized Logistic Regression for Predicting Asthma Severity.

				95% CI			
Variable	β (Coefficient)	SE (β)	OR	Lower	Upper	χ^2^	*p*-Value
Periostin (pg/mL)	0.030	0.054	1.030	0.930	1.176	0.281	0.5963
Age (years)	0.067	0.027	1.069	1.017	1.138	7.031	0.0080
Gender (Female vs. Male)	−0.060	0.619	0.942	0.267	3.315	0.009	0.9249
BMI (kg/m^2^)	−0.080	0.066	0.923	0.800	1.051	1.442	0.2298
NLR	−1.035	0.696	0.355	0.071	1.347	2.265	0.1323
PLR	0.001	0.010	1.001	0.981	1.021	0.003	0.9547
SII	0.004	0.003	1.004	0.999	1.010	2.166	0.1410
Eosinophilia (Yes)	1.328	0.759	3.775	0.881	21.080	3.175	0.0748

pg/mL: picograms per milliliter; BMI: Body mass index; kg/m^2^: kilogram per square meter; NLR: Neutrophil-to-Lymphocyte ratio; PLR: Platelet-to-Lymphocyte Ratio; SII: Systemic Immune-Inflammation Index; CI: Confidence Interval.

## Data Availability

All data generated or analyzed during this study are included in this published article and are available from the corresponding author upon reasonable request.

## Supplementary Materials

The following supporting information can be downloaded at https://www.mdpi.com/article/10.3390/diagnostics15233028/s1, Table S1: Calibration and Internal Validation Metrics (Periostin-Only Model) for Asthma; Table S2: Variable Importance from Gradient Boosting Model (Stability Analysis) for Asthma; Table S3: Calibration and Internal Validation Metrics (Periostin-Only Model) for Asthma Severity; Table S4: Variable Importance from Gradient Boosting Model (Stability Analysis) for Asthma Severity.

## Abbreviations

The following abbreviations are used in this manuscript:

LMICs	Low- and Middle-Income Countries
DALYs	Disability-Adjusted Life Years
ECM	Extracellular Matrix
IL	Interleukin
TGF-β	Transforming Growth Factor Beta
GINA	Global Initiative for Asthma
BMI	Body Mass Index
NAEPP	National Asthma Education and Prevention Program
ATS	American Thoracic Society
ELISA	Enzyme-Linked Immunosorbent Assay
NLR	Neutrophil-to-Lymphocyte Ratio
PLR	Platelet-to-Lymphocyte Ratio
OR	Odds Ratios
CI	Confidence Intervals
ROC	Receiver Operating Characteristic
AUC	Area Under the Curve
PCA	Principal Component Analysis
ACT	Asthma Control Test
FVC	Forced Vital Capacity
FEV_1_	Forced Expiratory Volume in 1 Sec
AEC	Absolute Eosinophil Count
ANC	Absolute Neutrophil Count
ALC	Absolute Lymphocyte Count
FeNO	Fractional Exhaled Nitric Oxide
SII	Systemic Immune-Inflammation Index
DCA	Decision Curve Analysis
GBM	Gradient Boosting Machine
MAE	Mean Absolute Error
