# Supplementary material for: Unsupervised Phenotyping of Asthma: Integrating Serum Periostin with Clinical and Inflammatory Profiles
Source: Diagnostics (Basel). 2025 Nov 27;15(23):3028. doi: 10.3390/diagnostics15233028 (PMC12691264; doi:10.3390/diagnostics15233028)
Supplement: Supplementary file 1 [file diagnostics-15-03028-s001.zip › diagnostics-3972193-supplementary.pdf]

diagnostics

MDPI

Article

Unsupervised Phenotyping of Asthma: Integrating Serum Periostin with Clinical and Inflammatory Profiles: Supplementary File

Sukanya Ravindran [1,*], Mohammed Kaleem Ullah [2,*], Medha Karnik [2], Mandya Venkateshmurthy Greeshma [1,2], Nidhi Bansal [1], Shreedhar Kulkarni [1], Rekha Vaddarahalli ShankaraSetty [1], SubbaRao V. Madhunapantula [2], Jayaraj Biligere Siddaiah [1], Sindaghatta Krishnarao Chaya [1], Komarla Sundararaja Lokesh [1], Swaroop Ramaiah [3], Sachith Srinivas [4], Vikhnesh Padmakaran [5], Malavika Shankar [6], Ashwaghosha Parthasarathi [7], Padukudru Anand Mahesh [1,†]

1 Department of Respiratory Medicine, JSS Medical College, JSS Academy of Higher Education and Research, Mysuru 570015, India
2 Center of Excellence in Molecular Biology and Regenerative Medicine (CEMR) Laboratory (DST-FIST Supported Center and ICMR Collaborating Center of Excellence – ICMR-CCoE), Department of Biochemistry (DST-FIST Supported Department), JSS Medical College, JSS Academy of Higher Education & Research (JSS AHER), Mysuru 570015, Karnataka, India
3 Hospitalist, Thomas Memorial Hospital, WVU Medicine, South Charleston, WV, USA
4 SHO, Respiratory Medicine, Barnsley NHS Foundation Trust, South Yorkshire, United Kingdom
5 Foundation Year 2, General Medicine, Barnsley NHS Foundation Trust, South Yorkshire, United Kingdom
6 Bridgeport Hospital, Yale New Haven Health, Bridgeport, CT, USA
7 Rutgers RWJ Barnabas Center for Climate, Health and Healthcare, New Brunswick, NJ, USA

*These authors contributed equally

†Correspondence:
Professor Padukudru Anand Mahesh
Department of Respiratory Medicine,
JSS Medical College, JSSAHER,
Mysore, Karnataka, India.
Direct line: +91-9448044003
Email: pamahesh@jssuni.edu.in

Name	Email	ORCID
Sukanya Ravindran	sukanya.hema29may@gmail.com	0009-0002-6507-8098
Mohammed Kaleem Ullah	Ka7eem@gmail.com	0000-0001-8470-3114
Medha Karnik	medhakarnik07@gmail.com	0000-0002-1591-8004
Mandya Venkateshmurthy Greeshma	greeshmagreekz27@gmail.com	0000-0003-2236-4588
Nidhi Bansal	dr.nidzz2117@gmail.com	0009-0003-8665-1313
Shreedhar Kulkarni	shreedhar.k.kulkarni@gmail.com	0009-0009-0147-8376
Rekha Vaddarahalli ShankaraSetty	rekha.setty23@gmail.com	0009-0008-6781-3627
SubbaRao V. Madhunapantula	mvsstsubbarao@jssuni.edu.in	0000-0001-9167-9271
Jayaraj Biligere Siddaiah	bsjayaraj@jssuni.edu.in	0000-0001-6055-4580
Sindaghatta Krishnarao Chaya	chaya.sindaghatta@gmail.com	0000-0002-4898-9466
Komarla Sundararaja Lokesh	lokeshpulmo@gmail.com	0000-0001-5651-1123
Swaroop Ramaiah	swaroop2408@gmail.com	0009-0007-9105-9970
Sachith Srinivas	sachithgowda043@gmail.com	0000-0002-8238-4903

Vikhnesh Padmakaran	drvikhneshpadmakaran@gmail.com	0009-0002-8510-1208
Malavika Shankar	malavika18.shankar@gmail.com	0000-0003-0462-3988
Ashwaghosha Parthasarathi	ashwa.partha@gmail.com	0000-0002-7270-0247
Padukudru Anand Mahesh	pamahesh@jssuni.edu.in	0000-0003-1632-5945

This table reports calibration and internal validation statistics for the periostin-only Firth logistic regression model used to discriminate asthma vs. healthy controls. Metrics were generated using 1,000-bootstrap resampling (rms package). Calibration slope, intercept, discrimination index (Dxy), and calibration error (Emax, MAE) quantify how well predicted probabilities match observed asthma outcomes (Table S1).

Table S1. Calibration, and Internal Validation Metrics (Periostin-Only Model) for Asthma

Metric	Value	Interpretation
Calibration slope (bootstrap-corrected)	0.883	Slight overfitting, acceptable
Calibration intercept	−0.005	Near-perfect alignment
Dxy (index.orig)	0.9749	Equivalent to c-statistic ~0.987
Dxy (optimism-corrected)	0.9506	Strong discrimination
R^2 (optimism-corrected)	0.788	Good explanatory power
Emax (Calibration error)	0.217	Acceptable
MAE (from calibration)	0.042	Good agreement between predicted/observed

Abbreviations: Dxy: Somers' D rank-correlation index; R^2: Coefficient of determination; Emax: Maximum absolute calibration error; MAE: Mean absolute error

This table shows the relative importance of predictors from a gradient boosting machine (GBM) model built to evaluate internal stability of the asthma diagnosis model. Repeated 10-fold cross-validation (caret package) was performed. Higher percentages indicate stronger contribution to distinguishing asthma vs. controls (Table S2).

Table S2. Variable Importance from Gradient Boosting Model (Stability Analysis) for Asthma

Predictor	Relative Importance (%)
Periostin	100.0
BMI	3.38
Pre-FEV_1 (% predicted)	2.33
Age	0.97
Gender	0.00
Smoker	0.00

Abbreviations: FEV_1: Forced Expiratory Volume in 1 second; BMI: Body Mass Index

This table provides calibration and validation statistics for the periostin-only model used to classify moderate vs. severe asthma. Metrics were obtained using 1,000-bootstrap resampling. Results show poor model discrimination and weak calibration, indicating periostin is not a reliable predictor of asthma severity (Table S3).

Table S3. Calibration, and Internal Validation Metrics (Periostin-Only Model) for Asthma Severity

Metric	Value	Interpretation
Calibration slope (bootstrap-corrected)	0.571	Poor calibration; underfitting likely
Calibration intercept	0.005	Nearly unbiased overall prediction
Dxy (index.orig)	0.1246	Weak discrimination (AUC ≈ 0.562)
Dxy (optimism-corrected)	0.1058	Very weak discrimination
R^2 (optimism-corrected)	−0.0114	No explained variance
Emax (Calibration error)	0.378	High calibration error
MAE (from calibration)	0.069	Moderate miscalibration

Abbreviations: Dxy: Somers' D rank-correlation index; R^2: Coefficient of determination; Emax: Maximum absolute calibration error; MAE: Mean absolute error

This table presents predictor importance from a GBM model assessing internal stability of the asthma severity model (moderate vs. severe). Repeated 10-fold cross-validation was used. Age and systemic inflammatory mark ers were the strongest contributors, while periostin had relatively low predictive value (Table S4).

Table S4. Variable Importance from Gradient Boosting Model (Stability Analysis) for Asthma Severity

Predictor	Relative Importance (%)
Age in years	100.000
SII	69.055
BMI	61.115
PLR	52.789
Periostin	36.077
NLR	33.176
Gender (Male)	2.825
Eosinophilia (Yes)	0.000

Abbreviations: SII: Systemic Immune-Inflammation Index; BMI: Body Mass Index; NLR: Neutrophil-to-Lymphocyte Ratio; PLR: Platelet-to-Lymphocyte Ratio
